# Supplementary material for: SCARB2 drives hepatocellular carcinoma tumor initiating cells via enhanced MYC transcriptional activity
Source: Nat Commun. 2023 Sep 22;14:5917. doi: 10.1038/s41467-023-41593-z (PMC10517016; doi:10.1038/s41467-023-41593-z)
Supplement: Supplementary file 1 — Supplementary information [file 41467_2023_41593_MOESM1_ESM.pdf]

Supplementary information

SCARB2 drives hepatocellular carcinoma tumour initiating cells via enhanced MYC transcriptional activity

Feng Wang[1,6], Yang Gao[1,2,6], Situ Xue[1,6], Luyao Zhao[1], Huimin Jiang[1], Tingting Zhang[1], Yunxuan Li[1], Chenxi Zhao[1], Fan Wu[3], Tana Siqin[3], Ying Liu[1], Jie Wu[1], Yechao Yan[1], Jian Yuan[4,5]*, Jian-dong Jiang[1]* and Ke Li[1]*

Supplementary information includes six Supplementary Figures and one Supplementary Tables.

Supplementary Figure 1. *SCARB2* deletion decreased the proliferation, invasion and tumorsphere formation of HCC cells.

Supplementary Figure 2. *SCARB2* deletion reduces the tumour growth and metastasis.

Supplementary Figure 3. The effects of *Scarb2* deletion on the expression of MYC target genes.

Supplementary Figure 4. SCARB2 disturbs HDAC3-mediated MYC acetylation.

Supplementary Figure 5. SPR assay examined the binding abilities of 10 candidate SCARB2 inhibitors.

Supplementary Figure 6. Effects of PMB on HCC mice.

Supplementary Table 1. Clinical characteristics of patients in this study.

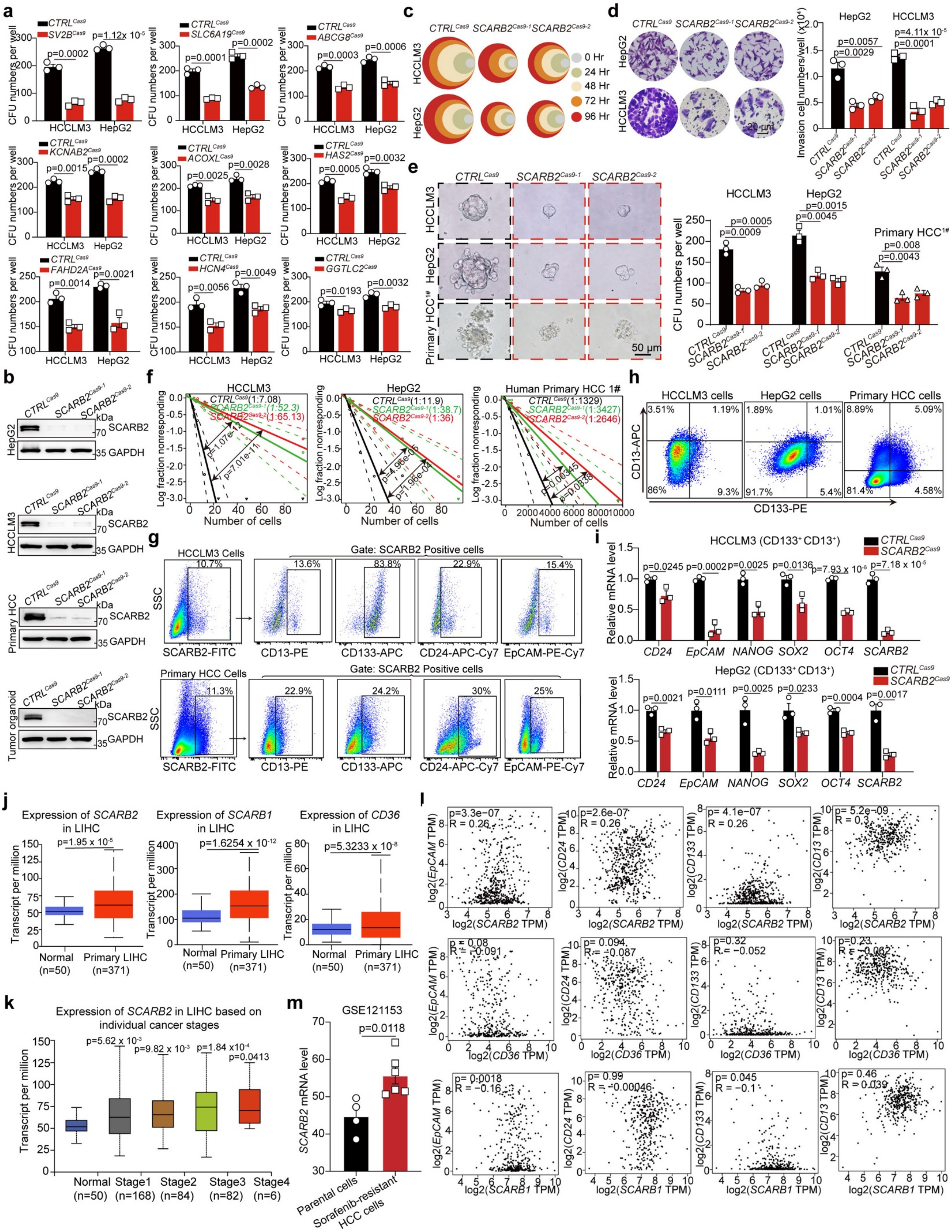

Supplementary Figure 1. ***SCARB2*** **deletion decreased the proliferation, invasion and tumorsphere**

formation of HCC cells.

(a) The effect of indicated gene knockout on the sphere-forming ability of HCC cells. **(b)** SCARB2 expression in the indicated groups were detected by western blotting. **(c)** Relative viability of HCCLM3 and HepG2 cells with or without *SCARB2* deletion at the indicated times was measured by a CCK-8 assay. The colors represent different time points; the diameter indicates the relative cell viability. **(d)** The effect of *SCARB2* knockout on the invasion of HepG2 and HCCLM3 cells. **(e)** Representative images and quantification of tumorspheres formed by HCCLM3, HepG2 or primary HCC cells with or without *SCARB2* knockout. **(f)** The frequency of tumor-initiating cells of HCC cells with or without *SCARB2* knockout was detected by *in vitro* limiting-dilution assays (n = 10 per group). (**g**) Flow cytometric analysis for detecting CD13, CD133, CD24, or EpCAM expression in SCARB2 positive cells from HCCLM3 cells or Primary HCC cells. **(h)** $CD133^{+}CD13^{+}$ and $CD133^{-}CD13^{-}$ subpopulations were sorted by flow cytometry from indicated cells. **(i)** Real-time PCR analysis of stem marker genes and stem transcription factor expression in $CD133^{+}CD13^{+}$ and $CD133^{-}CD13^{-}$ HCCLM3 cells. **(j)** Differential expression of *SCARB2*, *SCARB1* and *CD36* in primary HCC tissue samples (n=371) and normal liver tissue samples (n=50) were obtained and plotted using UALCAN platform [1] (https://ualcan.path.uab.edu/analysis.html). The center line indicates the median value**;** bounds of box = upper and lower quartiles**;** minima: bottom whiskers; maxima: top whiskers; (**k**) Expression of *SCARB2* in HCC based on individual cancer stages were obtained and plotted using UALCAN platform [1] (https://ualcan.path.uab.edu/analysis.html). The center line indicates the median value, whiskers= min to max. Normal liver tissue samples (n=50); Stage 1 (n=168); Stage 2 (n=84); Stage 3 (n=82); Stage 4 (n=6); The center line indicates the median value**;** bounds of box = upper and lower quartiles**;** minima: bottom whiskers; maxima: top whiskers; **(l)** Pearson's correlation between *SCARB2*, *SCARB1* or *CD36* expression with the known CSC markers gene (*CD133*, *EpCAM*, *CD24*, or *CD13*) was analyzed on GEPIA [2] (http://gepia.cancer-pku.cn). Transcript per million, TPM. Each data point represents the value from an individual patient. **(m)** The expression of *SCARB2* in sorafenib-resistant HCC cells (n=6) and their parental cells (n=4) from the GSE121153 dataset. **(a, b, c, d, e, g, h, i)** n = 3 biological repeats. Statistical significance was calculated by **(a, d, e, i, m)** two-tailed Student's t test; **(f)** one-sided extreme limiting dilution analysis. **(j, k)** Welch's T-test. **(l)** two-sided Pearson's correlation test; Data are presented as means ± S.E.M. Source data are provided as a Source Data file.

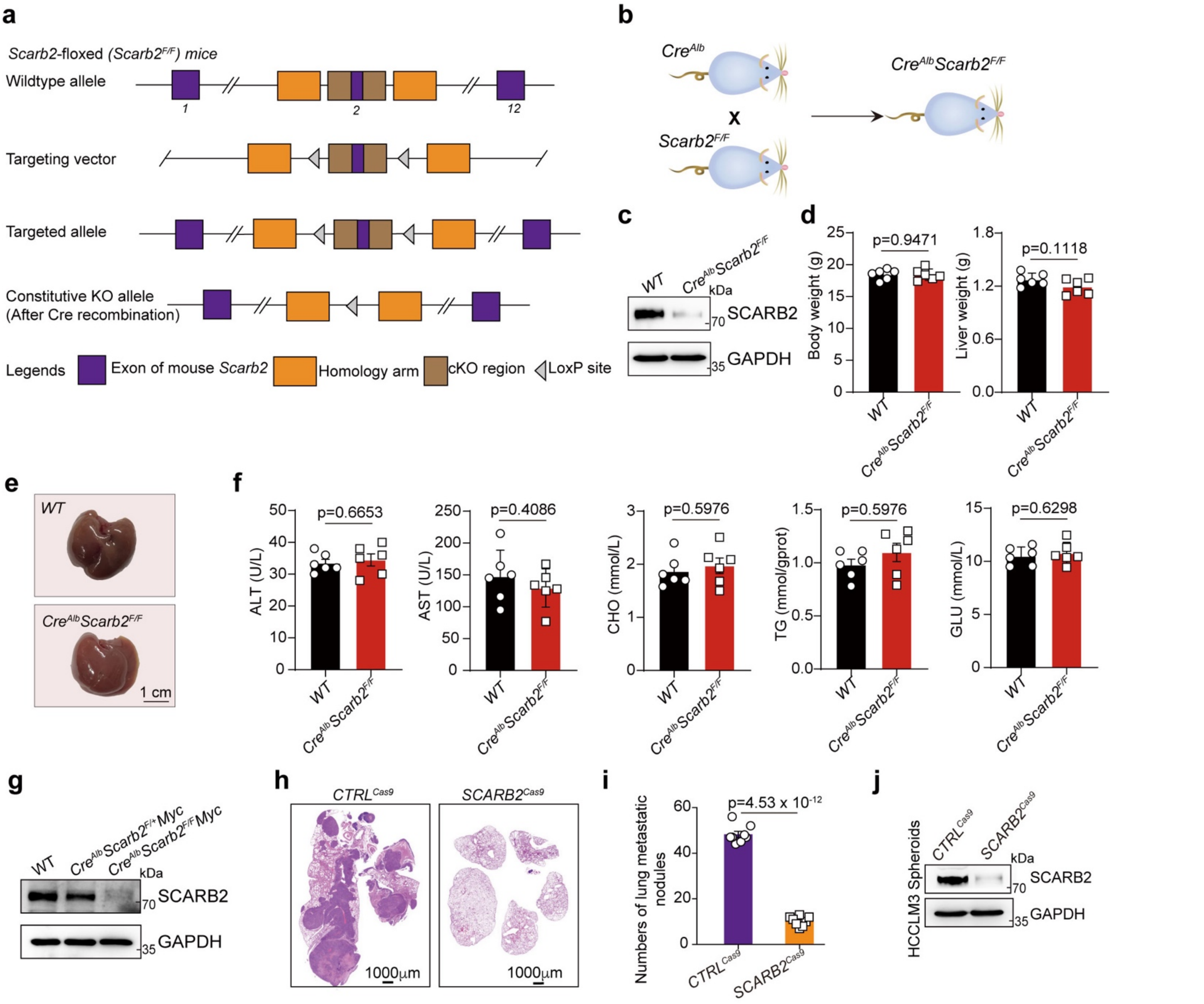

Supplementary Figure 2. *SCARB2* deletion reduces the tumour growth and metastasis.

(a-b) The generating scheme used to generate mice with *SCARB2* targeting knockout. **(c)** The SCARB2 expression in $Cre^{Alb}Scarb2^{F/F}$ mice and WT mice. The body/liver weight **(d)**, representative liver pictures **(e)**, serum ALT, AST, CHO, GLU, or TG levels (**f**) of 10-week–old $Cre^{Alb}Scarb2^{F/F}$ mice (n=6 mice) and WT mice (n=6 mice). Data are represented as means ± S.E.M. Statistical significance was determined by two-tailed Student's t test. (**g**) The SCARB2 expression in indicated mice were detected by western blotting. Data are representative images from three independent experiments. **(h)** Representative histological (H&E staining) images of lung metastatic tumors by in an *in vivo* metastasis assay. The number of metastatic lung foci detected in each group and histological staining were analyzed (n=8 mice per group). **(i)** Numbers of metastatic lung foci in the indicated mice (n=8 mice per group). Data are represented as means ± S.E.M. Statistical significance was determined by two tailed Student's t test. (**j**) SCARB2 expression in the indicated groups were detected by

western blotting. The data are presented as representative from 3 independent experiments. **(c, g, j)** n = 3 biological repeats. Statistical significance was calculated by **(d, f, i)** two-tailed Student's t test; Data are presented as means ± S.E.M. Source data are provided as a Source Data file.

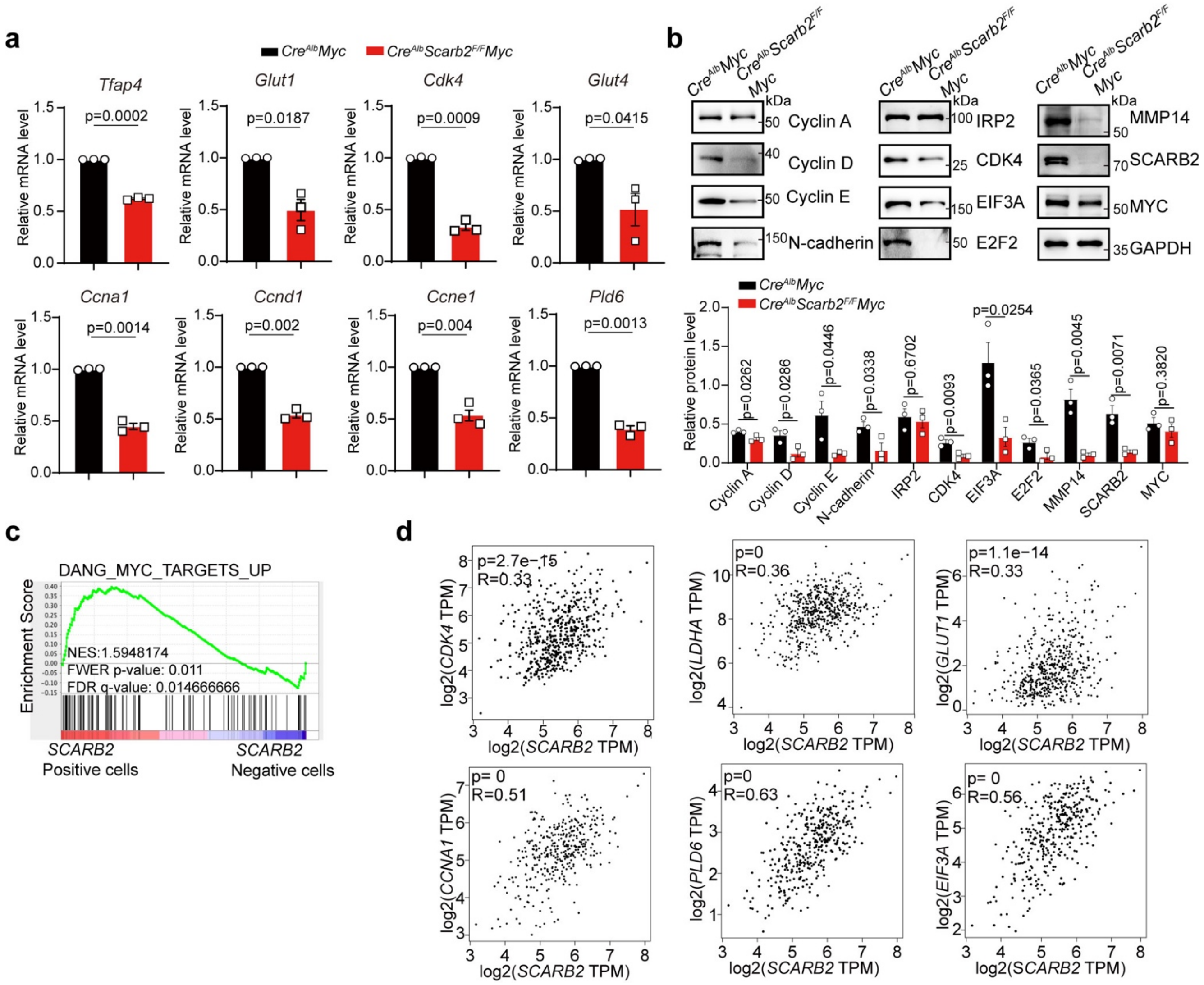

Supplementary Figure 3. The effects of *Scarb2* deletion on the expression of MYC target genes.

(a) The mRNA levels of *MYC* target genes in HCC cells from indicated mice were detected by Real-time PCR. **(b)** Protein levels of *MYC* target genes in the indicated mice (n=3 per group) were detected by western blotting. The samples were derived from the same experiment and that gels/blots were processed in parallel. **(c)** GSEA results showing the enrichment of MYC target genes in *SCARB2* positive cells and *SCARB2* negative cells. P-value was determined by one-sided permutation test. Statistical adjustments were made for multiple comparisons. **(d)** Pearson's correlation between SCARB2 with MYC target genes *CDK4, LDHA, GLUT1, CCNA1, PLD6,* or *EIF3A* using samples from GEPIA [2] (http://gepia.cancer-pku.cn). Each data point represents the value from an individual patient. **(a, b)** n = 3 biological repeats. Statistical significance was calculated by **(a, b)** two-tailed Student's t test; **(d)** two-sided Pearson's correlation test; Data are presented as means ± S.E.M. Source data are provided as a Source Data file.

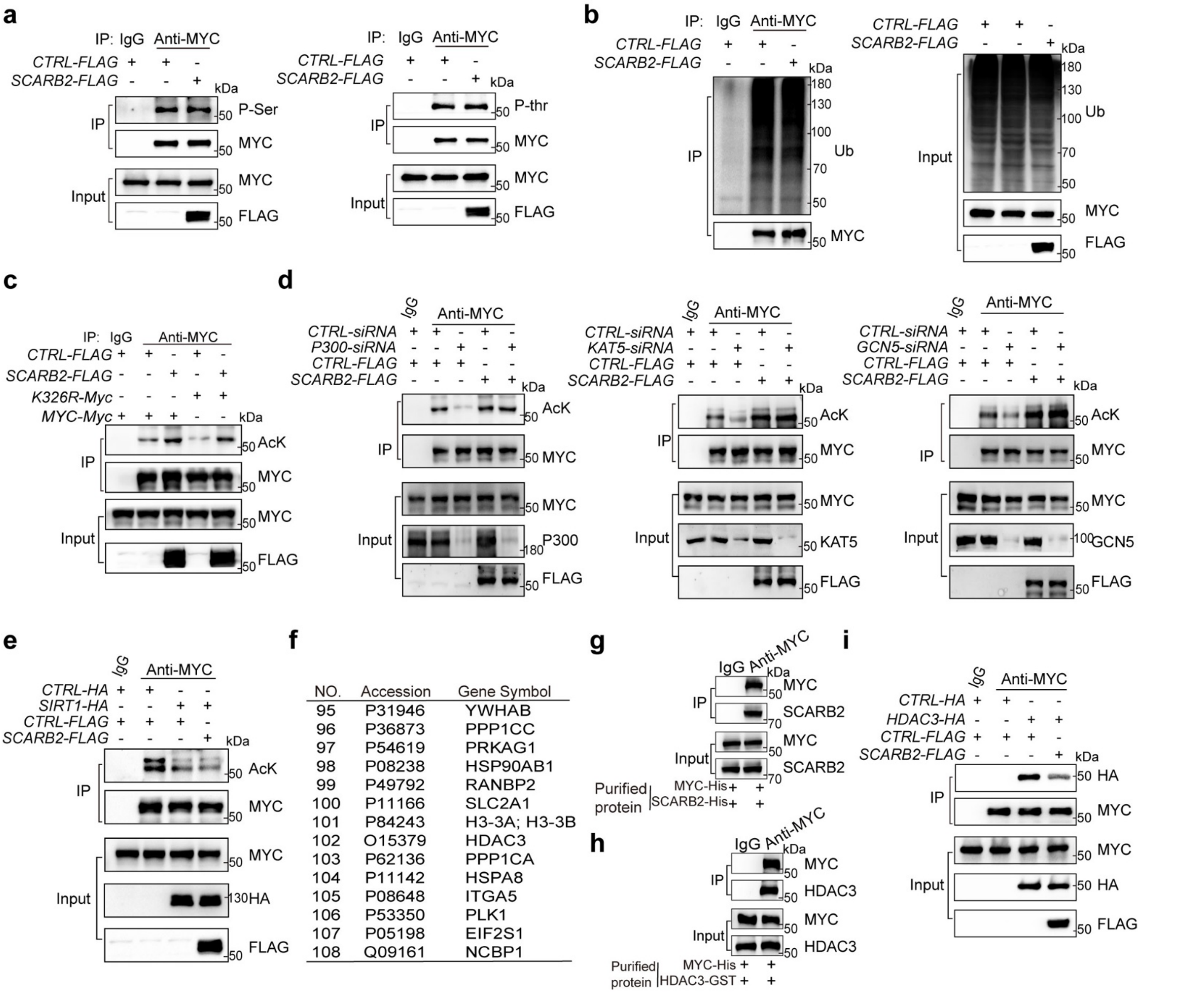

NO.	Accession	Gene Symbol
95	P31946	YWHAB
96	P36873	PPP1CC
97	P54619	PRKAG1
98	P08238	HSP90AB1
99	P49792	RANBP2
100	P11166	SLC2A1
101	P84243	H3-3A; H3-3B
102	O15379	HDAC3
103	P62136	PPP1CA
104	P11142	HSPA8
105	P08648	ITGA5
106	P53350	PLK1
107	P05198	EIF2S1
108	Q09161	NCBP1

Supplementary Figure 4. SCARB2 disturbs HDAC3-mediated MYC acetylation.

(a) The effects of *SCARB2* overexpression on the serine and threonine phosphorylation of MYC are assessed by Co- Immunoprecipitation analyses. **(b)** The effects of *SCARB2* overexpression on the ubiquitination of MYC are assessed by Co-Immunoprecipitation analyses. **(c)** Effects of SCARB2 on the acetylation of *MYC K326R* mutants. HEK 293T cells were transfected with the indicated mutants of MYC, and acetylation of MYC was detected by Co-IP. **(d)** The effects of *SCARB2* overexpression on the acetylation of MYC in HepG2 cells with or without *p300*, *KAT5*, or *GCN5* depletion. HepG2 cells were transfected with the indicated plasmids or siRNAs, and the acetylation of MYC was detected by Co-IP assays. **(e)** The effects of *SCARB2* overexpression in HepG2 cells on the acetylation of MYC with or without *SIRT1* overexpression. HepG2 cells were transfected with the indicated plasmids, and the acetylation of MYC was detected by Co-IP assays. **(f)** MS analyzed the interaction proteins of SCARB2 in HCCLM3 cells. **(g)** *In vitro* interaction assays detect the direct interaction of MYC and

SCARB2. **(h)** *In vitro* interaction assays detect the direct interaction of MYC and HDAC3. **(i)** *SCARB2* overexpression decreased the interaction of HDAC3 and MYC. *CTRL*^{OE} and *SCARB2*^{OE} cell extracts were immunoprecipitated with an anti-MYC Ab and immunoblotted with an anti-HA Ab. **(a, b, c, d, e, g, h, i)** n = 3 biological repeats. Source data are provided as a Source Data file.

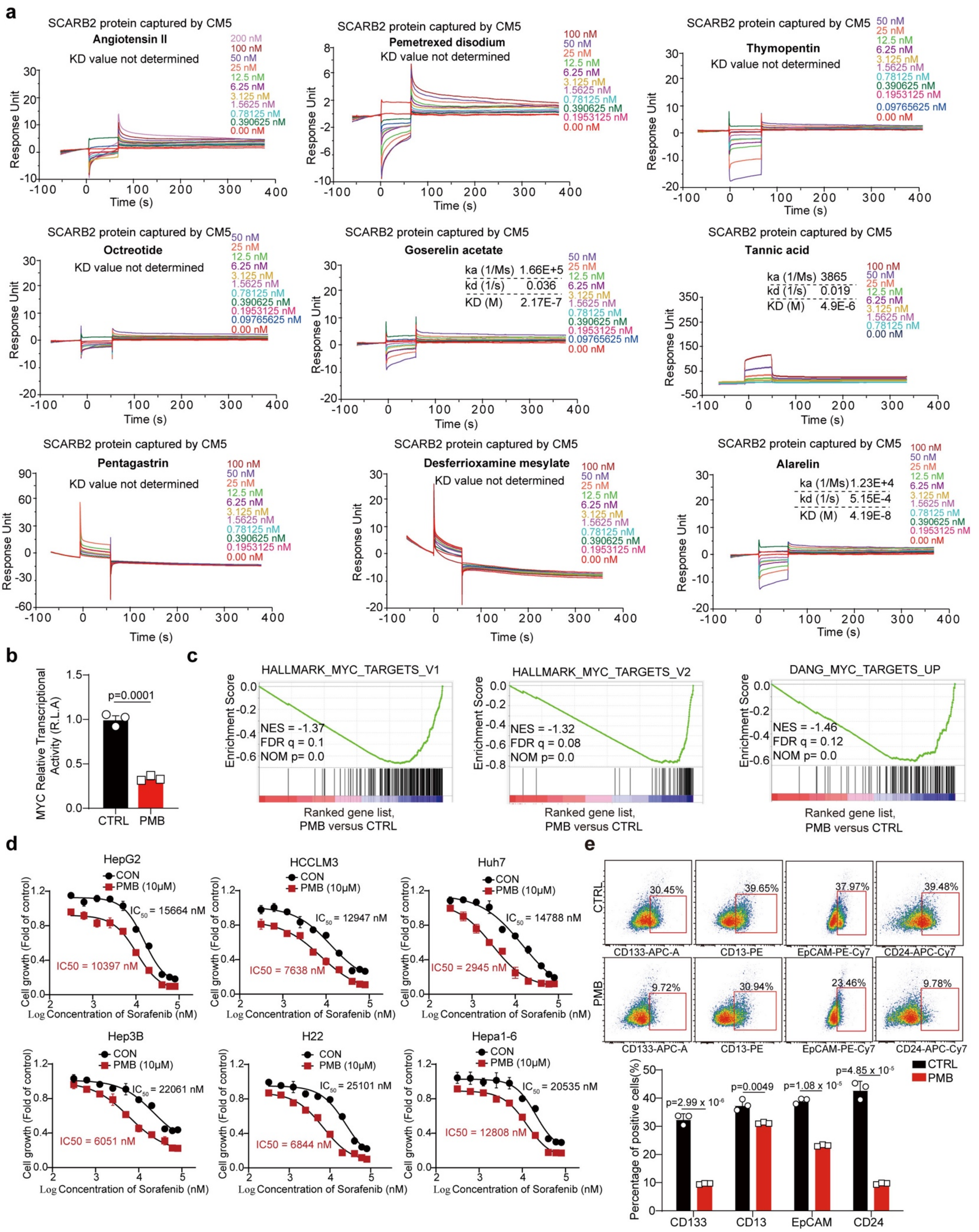

Supplementary Figure 5. SPR assay examined the binding abilities of 10 candidate SCARB2 inhibitors.

(a) Kinetic interaction of 10 candidated inhibitors and SCARB2 was determined by surface plasmon resonance (SPR) analyses. The indicated concentrations of SCARB2 inhibitors are passed over immobilized SCARB2 on CM5 chips. The affinity constants are evaluated using BIA evaluation software. **(b)** Transcriptional activity of MYC in HepG2 cells treated with or without PMB. Data are means ± S.E.M of 3 independent experiments. Statistical significance was determined by two-tailed Student's t test. **(c)** GSEA showed global downregulation of *MYC* target genes in PMB-treated vs. vehicle-treated HepG2 cells. P-value was determined by one-sided permutation test. Statistical adjustments were made for multiple comparisons. (**d**) Effects of PMB on sorafenib sensitivity in the indicated HCC cells. The data shown are a summary of the IC_{50} values for sorafenib in the indicated cell lines. **(e)** Flow cytometry analysis for detecting CD133, CD13, EpCAM, or CD24 expression in primary HCC cells with or without PMB treatment. Data are representative Flow cytometry plots of CD133, CD13, EpCAM, and CD24 staining. **(b, d, e)** n = 3 biological repeats. Statistical significance was calculated by **(b, e)** two-tailed Student's t test; Data are presented as means ± S.E.M. Source data are provided as a Source Data file.

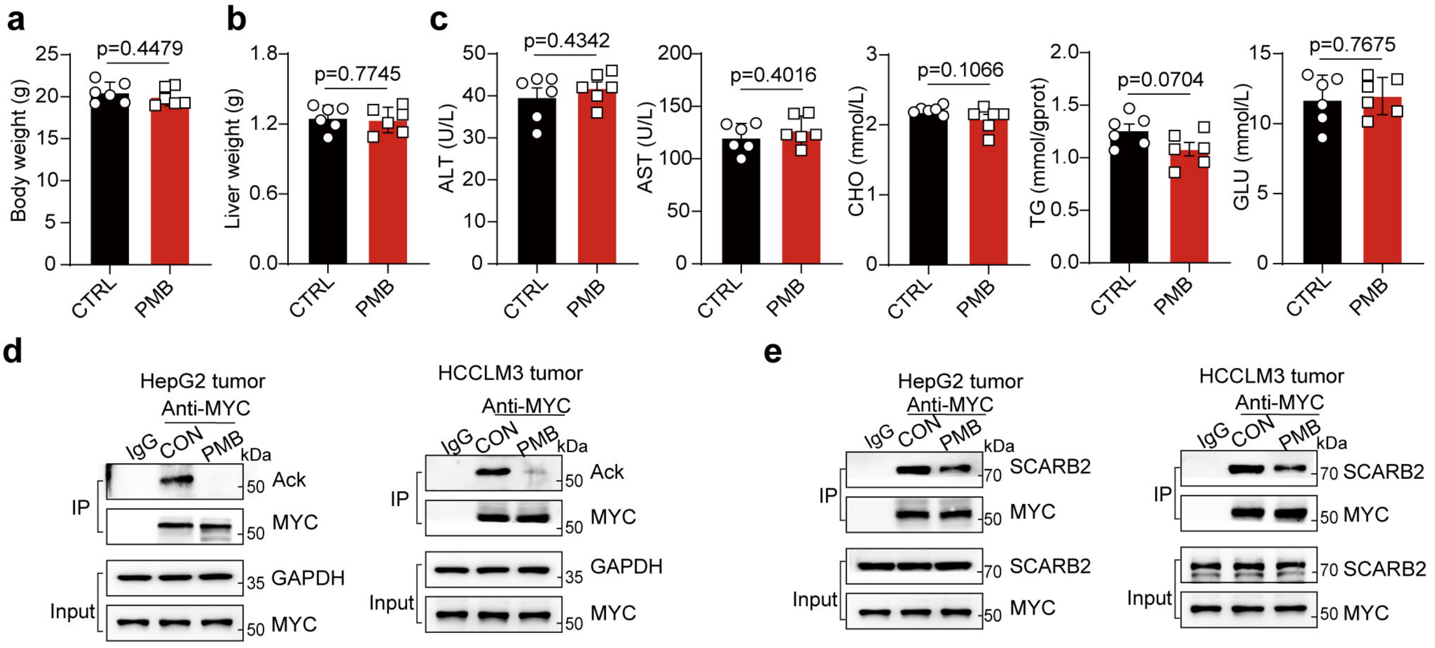

Supplementary Figure 6. Effects of PMB on HCC mice.

(a-c) The body weights **(a)**, liver weight **(b)**, serum ALT, AST, CHO, GLU, or TG levels **(c)** of HepG2 CDX mouse with or without PMB treatment (n = 6 mice per group). Data are represented as means ± S.E.M. Statistical significance was determined by two tailed Student's t test. **(d)** Effects of PMB on MYC acetylation *in vivo*. Tumor extracts from PMB-treated mice were immunoprecipitated with an anti-MYC Ab and immunoblotted with an anti-Ack ab. Data are representative images from three independent experiments. **(e)** PMB disrupted the SCARB2-MYC interaction *in vivo*. Tumor lysates from mice treated as indicated were subjected to IP with an anti-MYC Ab and immunoblotted with an anti-SCARB2 Ab. Data are representative images from three independent experiments. Source data are provided as a Source Data file.

Supplementary Table 1. Clinical characteristics of patients in this study.

Patient NO.	Gender	Age (years)	Subtype	Hep B	Hep C	Fatty liver	Diabetes	Hypertension
1	Male	67	Primary HCC	Positive	Negative	NO	NO	NO
2	Male	70	Primary HCC	Positive	Negative	NO	YES	YES
3	Male	55	Primary HCC	Positive	Negative	YES	NO	YES
4	Male	68	Primary HCC	Positive	Negative	NO	NO	NO
5	Female	54	Primary HCC	Positive	Negative	NO	NO	YES
6	Female	67	Primary HCC	Positive	Negative	NO	NO	YES
7	Female	70	Primary HCC	Positive	Negative	NO	NO	NO
8	Female	49	Primary HCC	Positive	Negative	NO	NO	NO

Supplementary References

1. Chandrashekar DS*, et al.* UALCAN: An update to the integrated cancer data analysis platform. *Neoplasia* **25**, 18-27 (2022).

2. Tang Z, Li C, Kang B, Gao G, Li C, Zhang Z. GEPIA: a web server for cancer and normal gene expression profiling and interactive analyses. *Nucleic Acids Res* **45**, W98-W102 (2017).
