# Supplementary material for: SCARB2 drives hepatocellular carcinoma tumor initiating cells via enhanced MYC transcriptional activity
Source: Nat Commun. 2023 Sep 22;14:5917. doi: 10.1038/s41467-023-41593-z (PMC10517016; doi:10.1038/s41467-023-41593-z)
Supplement: Supplementary file 2 — Reporting Summary [file 41467_2023_41593_MOESM2_ESM.pdf]

nature portfolio

Corresponding author(s): Ke Li, Jian-dong Jiang and Jian Yuan

Last updated by author(s): Aug 11, 2023

Reporting Summary

Nature Portfolio wishes to improve the reproducibility of the work that we publish. This form provides structure for consistency and transparency in reporting. For further information on Nature Portfolio policies, see our Editorial Policies and the Editorial Policy Checklist.

Statistics

For all statistical analyses, confirm that the following items are present in the figure legend, table legend, main text, or Methods section.

n/a	Confirmed	
☐	☒	The exact sample size (*n*) for each experimental group/condition, given as a discrete number and unit of measurement
☐	☒	A statement on whether measurements were taken from distinct samples or whether the same sample was measured repeatedly
☐	☒	The statistical test(s) used AND whether they are one- or two-sided<br>*Only common tests should be described solely by name; describe more complex techniques in the Methods section.*
☒	☐	A description of all covariates tested
☐	☒	A description of any assumptions or corrections, such as tests of normality and adjustment for multiple comparisons
☐	☒	A full description of the statistical parameters including central tendency (e.g. means) or other basic estimates (e.g. regression coefficient) AND variation (e.g. standard deviation) or associated estimates of uncertainty (e.g. confidence intervals)
☐	☒	For null hypothesis testing, the test statistic (e.g. *F*, *t*, *r*) with confidence intervals, effect sizes, degrees of freedom and *P* value noted<br>*Give P values as exact values whenever suitable.*
☒	☐	For Bayesian analysis, information on the choice of priors and Markov chain Monte Carlo settings
☒	☐	For hierarchical and complex designs, identification of the appropriate level for tests and full reporting of outcomes
☐	☒	Estimates of effect sizes (e.g. Cohen's *d*, Pearson's *r*), indicating how they were calculated

Our web collection on statistics for biologists contains articles on many of the points above.

Software and code

Policy information about availability of computer code

Data collection	Standard software and the respective analysis tools provided by manufacturers were listed in the methods (Olympus microsystems, Yena qTOWER3G PCR, GE BIAcore T200, Illumina NovaSeq 6000, Tanon 5200 chemiluminescent imaging system, Olympus DP72 microscope, Olympus Microsystems (Fv3000), BD FACS Verse, CytoFLEX flow cytometer.
Data analysis	Student's t-test, Pearson's correlation test and Kaplan-Meier analysis were done by using Prism GraphPad 9.0. The dissociation constant (KD) was calculated according to theBia Evaluation Software 4.1. All flow cytometry data were analyzed using FCS Express 6 or FlowJo 10.8.1 software. Immunohistochemistry analysis was performed by Image-Pro Plus 5.1. Western blots images were analyzed by Gel Pro Analyzer 3.2. Cell Ranger Software Suite (Version 3.0.2) was used to scRNA-seq analysis; Paired-end reads of CUT &Tag were aligned using Bowtie2 version 2.2.5. Quantitative proteomics raw data were processed with Proteome Discoverer 2.4.

For manuscripts utilizing custom algorithms or software that are central to the research but not yet described in published literature, software must be made available to editors and reviewers. We strongly encourage code deposition in a community repository (e.g. GitHub). See the Nature Portfolio guidelines for submitting code & software for further information.

Data

Policy information about availability of data

All manuscripts must include a data availability statement. This statement should provide the following information, where applicable:

- Accession codes, unique identifiers, or web links for publicly available datasets
- A description of any restrictions on data availability
- For clinical datasets or third party data, please ensure that the statement adheres to our policy

The RNA-seq, scRNA-seq and CUT&Tag data generated in this study have been deposited in the NCBI Gene Expression Omnibus (GEO) database under accession code GSE185844 and GSE207673. The mass spectrometry proteomics data generated in this study have been deposited to the ProteomeXchange Consortium (http://proteomecentral.proteomexchange.org) via the iProX partner repository under accession code PXD044544. The datasets from cancer genome atlas (TCGA) were analyzed using the UALCAN platform (https://ualcan.path.uab.edu/analysis.html). SCARB2 mRNA expression in sorafenib-resistant HCC cell and their parental cells was analyzed on the following accession code GSE121153. Pearson's correlation between SCARB2, SCARB1 or CD36 expression with the known CSC markers gene was analyzed on Gene Expression Profiling Interactive Analysis (GEPIA) database (http://gepia.cancer-pku.cn). The uncropped blot figures, and original data underlying Figs. 1–7 and Supplementary Figs. 1–6 are provided as a Source Data file. The remaining data are available within the Article, Supplementary Information or Source Data file. Source data are provided with this paper.

Human research participants

Policy information about studies involving human research participants and Sex and Gender in Research.

Reporting on sex and gender	Our analysis did not focus on identifying sex/gender-specific differences. We used HCC patient-derived tumor tissues to establish HCC PDX mouse models regardless of sex/gender.
Population characteristics	Tumor tissues were collected from patients with pathologically confirmed HCC undergoing surgery at the Cancer Hospital, Chinese Academy of Medical Sciences. The patients were Chinese with average age at resection was 62.5 years old. 4 patients were male and 4 female.
Recruitment	Patients were enrolled in by their oncologist at the Cancer Hospital, Chinese Academy of Medical Sciences. With informed consent from patients, and tumor specimens were collected after surgical removal of tumors.
Ethics oversight	The procedure was approved by the Ethics Committee of Cancer hospital, Chinese Academy of Medical Sciences.

Note that full information on the approval of the study protocol must also be provided in the manuscript.

Field-specific reporting

Please select the one below that is the best fit for your research. If you are not sure, read the appropriate sections before making your selection.

☒ Life sciences ☐ Behavioural & social sciences ☐ Ecological, evolutionary & environmental sciences

For a reference copy of the document with all sections, see nature.com/documents/nr-reporting-summary-flat.pdf

Life sciences study design

All studies must disclose on these points even when the disclosure is negative.

Sample size	For in vitro experiments, at least three biological replicates were achieved for most of the experiments, except for the CRISPR/Cas9. Such sample sizes are typical for the in vitro experiments and sufficient for a statistical analysis. Generally, we used n ≥ 6 mice per genotype and condition. For in vivo experiments, a sample size of n = 6-14 mice was used per experimental group. Sample size was determined based on our previous experience (Cancer Cell. 2017;31(5):697#710; Nat Commun. 2020;11 (1):6361; Sci Transl Med. 2021; 13 (586): eabb2914), which is sufficient to generate statistically significant results. No statistical method was used to predetermine sample size.
Data exclusions	No data were excluded from the analysis.
Replication	Most of the in vitro experiments were repeated independently at least three times except for those specifically indicated in the figure legends. Multiple mice (n = 6-14/group) were used for every in vivo experiment. All the attempts at replication were successful.
Randomization	For animal studies, the mice were earmarked before grouping and then were randomly separated into groups by an independent person before treatment. Cells were seeded identically at the oneset of the experiments and randomized into the various treatment groups prior to beginning of treatment protocols.
Blinding	Experimenters were blinded to group allocation for IHC staining and grading, quantification of tumorspheres numbers. All other experiments were performed in a non-blinded manner, because the experimental design was complicated, the researchers were limited, and blinding feasibility was poor.

Reporting for specific materials, systems and methods

We require information from authors about some types of materials, experimental systems and methods used in many studies. Here, indicate whether each material, system or method listed is relevant to your study. If you are not sure if a list item applies to your research, read the appropriate section before selecting a response.

Materials & experimental systems

n/a	Involved in the study
☐	☒ Antibodies
☐	☒ Eukaryotic cell lines
☒	☐ Palaeontology and archaeology
☐	☒ Animals and other organisms
☒	☐ Clinical data
☒	☐ Dual use research of concern

Methods

n/a	Involved in the study
☐	☒ ChIP-seq
☐	☒ Flow cytometry
☒	☐ MRI-based neuroimaging

Antibodies

Antibodies used

anti-GAPDH (ZSGB-BIO,TA-08, OT12D9, 1:2000), anti-MYC (D3N8F) (CST, #13987S, 1:1000), anti-MYC (CST, #9402S, 1:1000), anti-Max (S20) (CST, #4739, 1:1000), anti-acetylated lysine (CST, #9441, 1:1000), anti-HDAC3 (7G6C5) (CST, #3949, 1:1000), anti-SCARB2 (Abcam, ab176317, EPR12080, 1:1000), anti-GCN5 (Abcam, EPR21146, ab217876, 1:1000), anti-BRD4 (CST, #13440, Polyclonal,1:1000), anti-KAT5 (Abcam, ab151432, Polyclonal, 1:1000), anti-Cyclin A2 (E1D9T) (CST, #91500S, 1:1000), anti-Cyclin D (CST, #2922, 1:1000), anti-Cyclin E1 (D7T3U) (CST, #20808, 1:1000), anti-p300 (D8Z4E) (CST, #86377, 1:1000), anti-CDK4 (D9G3E) (CST, #12790, 1:1000), anti- N-Cadherin (D4R1H) (CST, #13116, 1:1000), anti-eIF3A (D51F4) (CST, #3411, 1:1000), anti-E2F2 (Abcam, ab138515, EPR8622, 1:1000), anti-IRP2 (D6E6W) (CST, #37135, 1:1000), anti-MMP14 (Abcam, ab51074, EP1264Y, 1:1000), anti-Lamin A/C (4C11) (CST, #4777, 1:1000), anti-Na, K-ATPase (D4Y7E) (CST, #99935, 1:1000), anti-IgG1 (G3A1) (CST, #5415, 1:2000), anti-Myc-tag (MBL, #562, 1:1000), anti-GFP-tag (MBL, #598, 1:1000), anti-DDDDK-tag (MBL, PM020, 1:1000), anti-HA-tag (MBL, #561, 1:1000), and anti-His-tag (MBL, PM032, 1:1000). For immunofluorescence and immunohistochemistry, the following antibodies were used: anti-MYC (R&D, AF3696, 1:100), anti-MYC (R&D, MAB3696, 1:100), anti-MYC (Novus, NB600-302, 1:100), anti-SCARB2 (Abcam, ab176317, EPR12080, 1:100), anti-HDAC3 (Novus, NB500-126, 1:100), Alexa Fluor 488 (Thermo Fisher, R37114, 1:200), Alexa Fluor 488 (Thermo Fisher, R37118, 1:200), Alexa Fluor 488 (Abcam, ab150173), Alexa Fluor 555 (Thermo Fisher, A-31572,1:200), Alexa Fluor 555 (Thermo Fisher, A-31570, 1:200), Alexa Fluor 555 (Thermo Fisher, A-21432, 1:200), Alexa Fluor 647 (Thermo Fisher, A-31571, 1:200), and Alexa Fluor 647 (Thermo Fisher, A-31573, 1:200). Flow cytometry: PE anti-human CD13(BioLegend, 301704, WM15, 1:100), APC/Cyanine7 anti-human CD24 (BioLegend, 311132, ML5, 1:100), APC anti-human CD133 (BioLegend, 394009, S16016B, 1:100), PE/Cyanine7 anti-human EpCAM (BioLegend, 324222, 9C4, 1:100), FITC anti-human EpCAM (BioLegend, 369813, C017-1A, 1:100), PE anti-mouse CD24 (Biolegend, 101807, M1/69, 1:100), PE/Cyanine7 anti-mouse CD133 (BioLegend, 141209, 315-2C11, 1:100), and APC anti-mouse EpCAM (BioLegend, 118214, G8.8, 1:100). For Cut & Tag, the following antibody was used: c-Myc Antibody (CST, #9402S, 3 µg/sample).

Validation

All antibodies are obtained from commercial sources, and validation was available from the websites of respective vendors;
anti-GAPDH, human and mouse, WB, (http://www.zsbio.com/product/TA-08); anti-c-Myc (D3N8F); anti-c-Myc, human and mouse, WB, IP, IF and ChIP, (https://www.cellsignal.cn/products/primary-antibodies/c-myc-n-myc-d3n8f-rabbit-mab/13987); anti-c-Myc,human and mouse, WB, IP and ChIP, (https://www.cellsignal.cn/products/primary-antibodies/c-myc-antibody/9402?N=4294956287&Ntt=9402s&fromPage=plp); anti-Max, human and mouse, WB, IP and IF, (https://www.cellsignal.cn/products/primary-antibodies/max-s20-antibody/4739?N=4294956287&Ntt=4739&fromPage=plp); anti-acetylated lysine, all species, WB, IP, IF and ChIP, (https://www.cellsignal.cn/products/primary-antibodies/acetylated-lysine-antibody/9441?site-search-type=Products&N=4294956287&Ntt=9441&fromPage=plp&_requestid=1588560); anti-HDAC3 (7G6C5), human and mouse, WB, IP and IF, (https://www.cellsignal.cn/products/primary-antibodies/hdac3-7g6c5-mouse-mab/3949?site-search-type=Products&N=4294956287&Ntt=3949%2C&fromPage=plp&_requestid=1591029); anti-SCARB2, human and mouse,WB, IP, IHC-P, (https://www.abcam.cn/limpii-antibody-epr12080-ab176317.html); anti-GCN5, human and mouse, WB, IP and CHIP, (https://www.abcam.cn/kat2a--gcn5-antibody-epr21146-chip-grade-ab217876.html); anti-BRD4, human, WB, IP and CHIP, (https://www.cellsignal.cn/products/primary-antibodies/brd4-e2a7x-rabbit-mab/13440?site-search-type=Products&N=4294956287&Ntt=13440&fromPage=plp&_requestid=1592552); anti-KAT5, human and mouse,WB, IHC https://www.abcam.cn/kat5--tip60-antibody-ab151432.html; anti-Cyclin A2 (E1D9T), human, WB, (https://www.cellsignal.cn/products/primary-antibodies/cyclin-a2-e1d9t-rabbit-mab/91500?site-search type=Products&N=4294956287&Ntt=91500s&fromPage=plp&_requestid=1595679); anti-Cyclin D1, human and mouse, WB and IP, (https://www.cellsignal.cn/products/primary-antibodies/cyclin-d1-antibody/2922?_=1665534634044&Ntt=2922,&tahead=true); anti-Cyclin E1 (D7T3U), human and mouse,WB, (https://www.cellsignal.cn/products/primary-antibodies/cyclin-e1-d7t3u-rabbit-mab/20808?site-search-type=Products&N=4294956287&Ntt=20808&fromPage=plp&_requestid=1601001); anti-p300 (D8Z4E), human and mouse, WB, IP and IHC, (https://www.cellsignal.cn/products/primary-antibodies/p300-d8z4e-rabbit-mab/86377?site-search-type=Products&N=4294956287&Ntt=86377&fromPage=plp&_requestid=1601197); anti-CDK4 (D9G3E), human and mouse, WB, IP and IHC, (https://www.cellsignal.cn/products/primary-antibodies/cdk4-d9g3e-rabbit-mab/12790?site-search-type=Products&N=4294956287&Ntt=12790&fromPage=plp&_requestid=1601750); anti- N-Cadherin (D4R1H), human and mouse,WB, IP and IHC, (https://www.cellsignal.cn/products/primary-antibodies/n-cadherin-d4r1h-xp-rabbit-mab/13116?site-search-type=Products&N=4294956287&Ntt=13116&fromPage=plp&_requestid=1601955); anti-eIF3A (D51F4), human and mouse, WB, IP and IF, (https://www.cellsignal.cn/products/primary-antibodies/eif3a-d51f4-xp-rabbit-mab/3411?site-search-type=Products&N=4294956287&Ntt=3411&fromPage=plp&_requestid=1602158); anti-E2F2, human , WB, Flow Cyt (Intra).(https://www.abcam.cn/products/primary-antibodies/e2f2-antibody-epr8622-ab138515.html); anti-IRP2 (D6E6W), human, WB, (https://www.cellsignal.cn/products/primary-antibodies/irp2-d6e6w-rabbit-mab/37135?site-search-

type=Products&N=4294956287&Ntt=37135&fromPage=plp&_requestid=1603190) ; anti-MMP14, Mouse, Rat, Human, IHC-IP, IP, Flow Cyt, WB, ICC/IF. (https://www.abcam.cn/products/primary-antibodies/mmp14-antibody-ep1264y-ab51074.html); anti-Lamin A/C ,Human, Mouse, Rat, Mk, WB, IP, IHC, IF, F. (https://www.cellsignal.cn/products/primary-antibodies/lamin-a-c-4c11-mouse-mab/4777); anti-Na, K-ATPase,Human, WB, IHC, IF. (https://www.cellsignal.cn/products/primary-antibodies/na-k-atpase-a1-d4y7e-rabbit-mab-bsa-and-azide-free/99935?site-search-type=Products&N=4294956287&Ntt=na-k-atp&fromPage=plp); anti-IgG1, all, IP, IHC,IF, F, CHIP, (https://www.cellsignal.cn/products/primary-antibodies/mouse-g3a1-mab-igg1-isotype-control/5415?site-search-type=Products&N=4294956287&Ntt=5415&fromPage=plp&_requestid=407315); anti-Myc, human and mouse, WB and IP, (https://ruo.mbl.co.jp/bio/e/dtl/A/index.html?pcd=562); anti-Myc, human and mouse, WB and IP, (https://ruo.mbl.co.jp/bio/e/dtl/A/?pcd=M047-3); anti-GFP, human and mouse, WB and IP, (https://ruo.mbl.co.jp/bio/e/dtl/A/?pcd=598); anti-GFP, human and mouse, WB and IP, (https://ruo.mbl.co.jp/bio/e/dtl/A/?pcd=M048-3); anti-DDDDK, human and mouse, WB and IP, (https://ruo.mbl.co.jp/bio/e/dtl/A/?pcd=PM020); anti-DDDDK, human and mouse, WB and IP, (https://ruo.mbl.co.jp/bio/e/dtl/A/?pcd=M185-3L); anti-HA, human and mouse, WB and IP, (https://ruo.mbl.co.jp/bio/e/dtl/A/?pcd=561); anti-HA, human and mouse, WB and IP, (https://ruo.mbl.co.jp/bio/e/dtl/A/?pcd=M180-3); anti-His, human and mouse, WB and IP, (https://ruo.mbl.co.jp/bio/e/dtl/A/?pcd=PM032); anti-His, human and mouse, WB and IP, (https://ruo.mbl.co.jp/bio/e/dtl/A/?pcd=D291-3); Immunofluorescence & anti-c-Myc, human and mouse, WB, IP, IHC,ICC/IF, (https://www.novusbio.com/products/c-myc-antibody_af3696); Immunofluorescence &anti-c-Myc, human and mouse, WB, IP, IHC,ICC/IF, (https://www.novusbio.com/products/c-myc-antibody-9e10_nb600-302); Immunofluorescence & anti-SCARB2, human and mouse,WB, IP, IHC-P, (https://www.abcam.cn/limpii-antibody-epr12080-ab176317.html); Immunofluorescence & anti-HDAC3, human and mouse, WB, IP, IHC,ICC/IF, (https://www.novusbio.com/products/hdac3-antibody_nb500-126); For Flow cytometry:PE anti-human CD13, human, IF, (https://www.biolegend.com/en-us/products/pe-anti-human-cd13-antibody-875); APC/Cyanine7 anti-human CD24, human, IF, (https://www.biolegend.com/en-us/products/apc-cyanine7-anti-human-cd24-antibody-12387); APC anti-human CD133, human, IF, (https://www.biolegend.com/en-us/products/apc-anti-human-cd133-antibody-18551); PE/Cyanine7 anti-human EpCAM, human, IF, (https://www.biolegend.com/en-us/products/pe-cyanine7-anti-human-cd326-epcam-antibody-8107); FITC anti-human EpCAM (https://www.biolegend.com/en-us/products/fitc-anti-human-cd326-epcam-antibody-14156); PE anti-mouse CD24 (https://www.biolegend.com/en-us/products/pe-anti-mouse-cd24-antibody-343); PE/Cyanine7 anti-mouse CD133 (https://www.biolegend.com/en-us/products/pe-cyanine7-anti-mouse-cd133-antibody-10193); APC anti-mouse EpCAM (https://www.biolegend.com/en-us/products/apc-anti-mouse-cd326-ep-cam-antibody-4974). Cut and tag : c-Myc Antibody, anti-c-Myc,human and mouse, WB, IP and ChIP, (https://www.cellsignal.cn/products/primary-antibodies/c-myc-antibody/9402?N=4294956287&Ntt=9402s&fromPage=plp);

Eukaryotic cell lines

Policy information about cell lines and Sex and Gender in Research

Cell line source(s)	HepG2, Huh7, Hep3B, Hepa1-6, H22, 293FT, and HEK 293T cells were purchased from Cell Resource Center, Peking Union Medical College. HCCLM3 cells were purchased from the China Center for Type Culture Collection (CCTCC).
Authentication	All the cell lines were recently authenticated by STR profiling.
Mycoplasma contamination	All cell lines were verified negative for mycoplasma contamination by MycoAlertTM Mycoplasma Detection Kit (Lonza, LT07-318).
Commonly misidentified lines (See ICLAC register)	No commonly misidentified cell lines were used.

Animals and other research organisms

Policy information about studies involving animals; ARRIVE guidelines recommended for reporting animal research, and Sex and Gender in Research

Laboratory animals	NOD-scid IL2Rgnull (NSG) mice (5-6 weeks old, male) were purchased from Shanghai Nan Fang Model Biotechnology Co. Ltd. (Shanghai, China). BALB/c nude mice (5-6 weeks old, female) and C57BL/6 mice (5-6 weeks old, male) were purchased from Beijing Hua Fu Kang Bioscience Co., Ltd. (Beijing, China). CreAlb (B6.Cg-Speer6-ps1Tg(Alb-cre)21Mgn/J) mice (5–6 weeks old, 1 male and 2 females; The Jackson Laboratory, 003574) were obtained from Cyagen Biosciences Inc. (Suzhou, China). Scarb2 knockout (Scarb2loxP/loxP, Scarb2F/F) (5–6 weeks old, 1 male and 2 females) mice were generated by Cyagen Biosciences Inc. (Suzhou, China). The gRNA for the mouse Scarb2 gene, the donor vector containing loxP sites, and Cas9 mRNA were coinjected into fertilized mouse eggs to generate offspring with targeted conditional knockout. Mice with hepatocyte-specific conditional Scarb2 knockout were generated by crossing Scarb2F/F mice with CreAlb mice. MycF/F (C57BL/6JSmoc-Igs2em1(CAG-LSL-Myc)Smoc)(5–6 weeks old, 1 male and 2 females, NM-KI-00039) mice were obtained from the Shanghai Research Center for Model Organisms (Shanghai, China). The conditional overexpression sequence with the CAG promoter-loxp-STOP-loxp-Myc-polyA structure was inserted into the H11 locus to establish the H11-LSL-Myc mouse model. For the establishment of PDX mouse models, NSG mice (5-6 weeks old, male) were used to establish.
Wild animals	The study did not involve wild animals.
Reporting on sex	Sex was not considered in this study.
Field-collected samples	No field-collected samples were used in this study.
Ethics oversight	All animal studies were approved by the Animal Experimentation Ethics Committee of the Chinese Academy of Medical Sciences (Permit No. IMB-20190423D702).

Note that full information on the approval of the study protocol must also be provided in the manuscript.

ChIP-seq

Data deposition

- [x] Confirm that both raw and final processed data have been deposited in a public database such as GEO.
- [x] Confirm that you have deposited or provided access to graph files (e.g. BED files) for the called peaks.

Data access links<br>*May remain private before publication.*	GSE207673:https://www.ncbi.nlm.nih.gov/geo/query/acc.cgi?acc=GSE207673<br>GSE185844: https://www.ncbi.nlm.nih.gov/geo/query/acc.cgi?acc=GSE185844
Files in database submission	IP-CTRL-cas9-1.bw, IP-SCARB2-ca9-1.bw, IP-CTRL-cas9-2.bw,IP-SCARB2-ca9-2.bw,IP-CTRL-cas9-3.bw,IP-SCARB2-ca9-3.bw, IP-CTRL-cas9-1_R1.fq, IP-CTRL-cas9-1_R2.fq, IP-CTRL-cas9-2_R1.fq, IP-CTRL-cas9-2_R2.fq,IP-CTRL-cas9-3_R1.fq, IP-CTRL-cas9-3_R2.fq,IP-SCARB2-ca9-1_R1.fq, IP-SCARB2-ca9-1_R2.fq, IP-SCARB2-ca9-2_R1.fq, IP-SCARB2-ca9-2_R2.fq, IP-SCARB2-ca9-3_R1.fq, IP-SCARB2-ca9-3_R2.fq, IP-IgG_R1.fq, IP-IgG_R2.fq
Genome browser session<br>(e.g. UCSC)	Homo_sapiens.GRCh38.89.chr

Methodology

Replicates	3 biological replicates each group
Sequencing depth	The total number of reads is 20M, the uniquely mapped reads is around 35 million, the length of reads is 150bp and they are paired-end.
Antibodies	ChIP grade antibodies specific to c-Myc (Cell Signaling Technology, #9402S, 3 ug/sample).
Peak calling parameters	Unambiguously mapped reads were retained for subsequent generation of binding profiles, heatmaps and calling of peaks. Mapped reads were used for peak calling with IgG sample as control and generation of bw files with MACS2, which were visualized using Integrative Genomics Viewer (IGV).
Data quality	To ensure date quality, we used the Q30, about 500 peaks are at FDR 5% and above 5-fold enrichment.
Software	The resulting fastp files were used for alignment with Bowtie2 version 2.2.5.

Flow Cytometry

Plots

Confirm that:

- [x] The axis labels state the marker and fluorochrome used (e.g. CD4-FITC).
- [x] The axis scales are clearly visible. Include numbers along axes only for bottom left plot of group (a 'group' is an analysis of identical markers).
- [x] All plots are contour plots with outliers or pseudocolor plots.
- [x] A numerical value for number of cells or percentage (with statistics) is provided.

Methodology

Sample preparation	About 1*106 single cell from primary liver cancer cells or HCC cell lines were suspended in PBS and incubated with relevant antibodies for 30min at room temperature .
Instrument	Data was acquired using BD FACS Verse or CytoFLEX flow cytometer.
Software	Data were analyzed with FCS EXPRESS or FlowJo 10.8.1 software.
Cell population abundance	Cell suspensions from HCC cell lines and primary human HCC cells were stained with PE anti-human CD13 and APC anti-human CD133, followed by being sorted CD13+CD133+ and CD13-CD133- with FACS Aria III (BD Bioscience).
Gating strategy	In the Fig 1g, Fig 1o, Fig 2r, and Supplementary Fig 5e, the gating strategy was as follow: FSC-A/SSC-A was used to gate live cells, then the CD133+, CD24+, EpCAM, or CD13+ cells were gated. In the Supplementary 1g , the gating strategy is FSC-A/SSC-A was used to gate live cells, SCARB2 positive cells were gated, then the CD133+, CD24+, EpCAM, or CD13+ cells were gated. In the Supplementary 1h, the gating strategy is FSC-A/SSC-A was used to gate live cells, and the CD133+ CD13+ cells were gated.

- [x] Tick this box to confirm that a figure exemplifying the gating strategy is provided in the Supplementary Information.
