# Supplementary material for: SCARB2 drives hepatocellular carcinoma tumor initiating cells via enhanced MYC transcriptional activity
Source: Nat Commun. 2023 Sep 22;14:5917. doi: 10.1038/s41467-023-41593-z (PMC10517016; doi:10.1038/s41467-023-41593-z)
Supplement: Supplementary file 3 — Description of Additional Supplementary Files [file 41467_2023_41593_MOESM3_ESM.pdf]

Title: Supplementary Data 1
Description: The information of human CRISPR/Cas9 metabolic gene knockout library.

Title: Supplementary Data 2
Description: The analysis of metabolic CRISPR library screen by MAGeCK.
